# *In vivo* imaging of mice infected with bioluminescent *Trypanosoma cruzi* unveils novel sites of infection

**DOI:** 10.1186/1756-3305-7-89

**Published:** 2014-03-03

**Authors:** Cristina Henriques, Andréa Henriques-Pons, Marcelo Meuser-Batista, Aline Salgado Ribeiro, Wanderley de Souza

**Affiliations:** 1 Laboratório de Ultraestrutura Celular Hertha Meyer, Instituto de Biofísica Carlos Chagas Filho, Universidade Federal do Rio de Janeiro, CCS-Bloco G, Ilha do Fundão, 21941-900 Rio de Janeiro-RJ, Brazil; 2 Instituto Nacional de Ciência e Tecnologia em Biologia Estrutural e Biomagens-INBEB, Janeiro-RJ, Brazil; 3 Nucleo de Biologia Estrutural e Biomagens, Universidade Federal do Rio de Janeiro-CENABIO, Janeiro-RJ, Brazil; 4 Fundação Oswaldo Cruz, Cruz- FIOCRUZ, Mato Grosso do Sul, Campo Grande-MS, Brazil; 5 Laboratório de Inovações em Terapias, Ensino e Bioprodutos, Instituto Oswaldo Cruz-Fundação Oswaldo Cruz, Janeiro-RJ, Brazil; 6 Departamento de Anatomia Patológica e Citopatologia, Instituto Fernandes Figueira-FIOCRUZ, Janeiro-RJ, Brazil; 7 Instituto Nacional de Metrologia, Qualidade e Tecnologia-Inmetro, Janeiro-RJ, Brazil

**Keywords:** *Trypanosoma cruzi*, Luciferase, Bioluminescence, Testicles, Rectum, Heart, Mice, Chagas disease

## Abstract

**Background:**

The development of techniques that allow the imaging of animals infected with parasites expressing luciferase opens up new possibilities for following the fate of parasites in infected mammals.

**Methods:**

D-luciferin potassium salt stock solution was prepared in phosphate-buffered saline (PBS) at 15 mg/ml. To produce bioluminescence, infected and control mice received an intraperitoneal injection of luciferin stock solution (150 mg/kg). All mice were immediately anesthetized with 2% isofluorane, and after 10 minutes were imaged. *Ex vivo* evaluation of infected tissues and organs was evaluated in a 24-well plate in 150 μg/ml D-luciferin diluted in PBS. Images were captured using the IVIS Lumina image system (Xenogen). Dissected organs were also evaluated by microscopy of hematoxylin-eosin stained sections.

**Results:**

Here we describe the results obtained using a genetically modified Dm28c strain of *T. cruzi* expressing the firefly luciferase to keep track of infection by bioluminescence imaging. Progression of infection was observed *in vivo* in BALB/c mice at various intervals after infection with transgenic Dm28c-luc. The bioluminescent signal was immediately observed at the site of *T. cruzi* inoculation, and one day post infection (dpi) it was disseminated in the peritoneal cavity. A similar pattern in the cavity was observed on 7 dpi, but the bioluminescence was more intense in the terminal region of the large intestine, rectum, and gonads. On 14 and 21 dpi, bioluminescent parasites were also observed in the heart, snout, paws, hind limbs, and forelimbs. From 28 dpi to 180 dpi in chronically infected mice, bioluminescence declined in regions of the body but was concentrated in the gonad region. *Ex vivo* evaluation of dissected organs and tissues by bioluminescent imaging confirmed the *in vivo* bioluminescent foci. Histopathological analysis of dissected organs demonstrated parasite nests at the rectum and snout, in muscle fibers of mice infected with Dm28c-WT and with Dm28c-luc, corroborating the bioluminescent imaging.

**Conclusion:**

Bioluminescence imaging is accurate for tracking parasites *in vivo*, and this methodology is important to gain a better understanding of the infection, tissue inflammation, and parasite biology regarding host cell interaction, proliferation, and parasite clearance to subpatent levels.

## Background

*Trypanosoma cruzi* is the etiological agent of Chagas disease, a debilitating illness that still causes 10,000 to 14,000 deaths per year and affects millions of people in Central and South America, where 8 to 10 million people are infected [1]. *T. cruzi* has a complex life cycle that involves the following three developmental stages: amastigote (intracellular replicative form in vertebrate host), epimastigote (extracellular replicative form found in the gut of invertebrate host), and trypomastigote (infective form for vertebrate hosts) [2]. It has been shown that *T. cruzi* is able to infect all nucleated mammalian cells in culture. *In vivo* it has been shown that even adipocytes can be infected by trypomastigotes [3]. However, cells of the reticuloendothelial, nervous and muscle systems appear to be the preferential ones in the experimental model and in *T. cruzi*-infected individuals [4,5].

For a more complete understanding of the tissue distribution of *T. cruzi* in the entire animal and the evolution of the infection during the course of analysis of new anti-parasite drugs, it is important to establish experimental models in which the parasites can be easily followed within the animal. Currently available chemotherapeutic agents for Chagas disease are nifurtimox and benznidazole. Although both are active in the acute phase, their efficacy is very limited during the chronic phase. In addition, both drugs demonstrate poor activity against many *T. cruzi* isolates, and have considerable side effects that can lead to the discontinuity of the therapy [6]. In recent years, several studies attempted to identify selective metabolic targets for new drugs, making this a very active area of research. Enzymes and metabolic pathways found by proteomic, transcriptomic, and metabolomic analysis [7-9], coupled with platforms of drug design, necessitate more advanced methods of screening compounds *in vivo*. Novel methodologies and technologies have been developed for *in vitro* screening of compounds with inhibitory activity against protozoa, such as viability and proliferative assays [10]. This includes the use of transgenic parasites expressing fluorescent proteins and/or luciferase, in conjunction with sensitive imaging systems and instruments.

The bioluminescent imaging represents an innovation in pre-clinical evaluation of drugs *in vivo* and in the follow-up of infection in small mammals. The foci of infection can be tracked throughout the body in real time, as long as the animal is alive. This noninvasive technology gives light to the new sites of infection that could not be observed easily through histological techniques and allows investigators to evaluate the dynamic of parasite distribution in the whole body, a methodology that does not require killing the animal to evaluate infected tissues. Some studies on standardization of this important technological tool, using *Trypanosoma cruzi*, *Leishmania sp*, and *Toxoplasma gondii*, have shown its application [11-14]. We previously described the use of bioluminescence imaging to follow the fate of *T. cruzi* in the invertebrate host [12]. In the present work, we explored, using a bioluminescent imaging system, the progression of the infection in mice infected with transgenic Dm28c-luc strain of *Trypanosoma cruzi* expressing luciferase. It was possible to monitor in real time the progression of infection in the same group of infected mice. This innovative technology enabled a better evaluation of the acute infection, including the discovery of tissues and organs with intense parasitism during the course of experimental infection up to the chronic phase of the disease. Bioluminescent imaging is a powerful tool for the evaluation of the effect of anti-parasite compounds on the evolution of ongoing biological processes in live mammals and other organisms.

## Methods

### Parasites

We used wild type Dm28c clone(Dm28c-WT) of *T. cruzi* and genetically modified parasites expressing luciferase (Dm28c-luc) (description below). Epimastigote forms of both strains were cultivated in liver infusion tryptose (LIT) medium supplemented with 10% fetal calf serum (FCS) at 28°C up to the logarithmic stage of growth [15]. Metacyclic trypomastigote forms were then obtained *in vitro* by subjecting *T. cruzi* epimastigotes from the late exponential growth phase (at cell density of 3 × 10^7^ cells/ml) to nutritional stress in triatomine artificial urine (TAU) medium (190 mM NaCl, 8 mM phosphate buffer, 17 mM KCl, 2 mM MgCl_2_, pH 6.0) for 2 hours. Metacyclic parasites were further cultivated for five days in TAU medium supplemented with amino acids and glucose (TAU3AAG) (TAU supplemented with 0.035% sodium bicarbonate, 10 mM L-proline, 50 mM sodium glutamate, 2 mM sodium L-aspartate, and 10 mM glucose) [16]. Metacyclic parasites were used to infect LLC-MK2 cells and trypomastigote forms released from cell cultures were used to infect mice.

Intracellular amastigotes and amastigotes were obtained from LLC-MK2 cells infected with trypomastigotes and cultivated for 5 to 10 days in RPMI 1640 medium supplemented with 5% FCS at 37°C in a 5% CO_2_ atmosphere.

Dm28c-luc trypomastigotes and/or amastigotes of cell culture, maintained in culture for 5 months without G418, were differentianted back to epimastigotes after incubation in LIT medium supplemented with 10% FCS at 28°C. Bioluminescence in epimastigotes were evaluated in a microplate reader Spectra Max2.

### Infection rate

LLC-MK2 cells were plated on 13 mm round glass coverslips in a 24 well microplate, maintained for 18 hours in RPMI 1640 medium with 5% FBS at 37°C in a 5% CO_2_ atmosphere. Afterwards, the cells were washed and allowed to interact with trypomastigotes of Dm28c-WT or Dm28c-luc with a parasite-host cell ratio of 10:1, in 200 μl of RPMI at 37°C in a 5% CO_2_ atmosphere. After 4 hours, cultures were washed to remove non-internalized parasites and maintained in RPMI 1640 medium with 5% FBS at 37°C in a 5% CO_2_ atmosphere for 24, 48 and 72 hours. Infected cells were fixed with Bouin, washed with 70% ethanol, water and stained with Giemsa. Subsequently, coverslips were dehydrated in acetone–xylol (100% acetone; 70% acetone – 30% xylol; 30% acetone–70% xylol; 100% xylol) and sealed with Entelan® (Merck). The percentage of infection and number of parasites per infected cells were quantified in at least 500 cells in a light microscope (Leica Microsystems). Two independent experiments were performed in triplicate.

### Animals and infection

Seven-week-old male BALB/c mice were obtained from the Animal Laboratory Breeding Center at Fundação Oswaldo Cruz (CECAL) and housed for 7 days in the Laboratory of Cellular Ultrastructure at UFRJ under environmental and sanitation conditions established in the guide for the Care and Use of Laboratory Animals (DHEW publication No. [NIH] 80–23). This project was approved by the Biophysics Institution Committee of Ethics in Animal Research (IBCCF106), according to resolution 196/96 of the National Health Council of the Brazilian Ministry of Health. Experimental groups were composed of five mice, which were intraperitoneally infected with 1 × 10^6^ or 1 × 10^5^ Dm28c-WT or Dm28c-luc parasites in 200 μl of RPMI medium. Parasitemia was determined in 5 μl of blood obtained from tail snips, according to the Pizzi-Brenner method [17].

### Expression of firefly luciferase (Fluc) in *Trypanosoma cruzi*

The luciferase gene was amplified by PCR using specific primers, the forward containing an XbaI site and the kozak sequence upstream from the start codon (italics), 5′-GC*TCTAGA GCCACC* ATGGAAGACGCCAAAAACATAAAG–3′ (F-luc), and the reverse primer containing an XhoI site (italic) 5′-CCG*CTCGAG* CGGTTACACGGCGATCTTTCC-3′ (R-luc), using the Tli DNA Polymerase (Promega) and the following PCR conditions: 94°C, 5 min; 94°C, 30 sec; 60°C, 30 sec; 72°C, 2 min, 30 cycles; 72°C, 10 min. The PCR product was cloned into the Zero Blunt TOPO PCR Cloning Kit (Invitrogen), digested from the TOPO vector, and subcloned into the integrative pTREX vector at the XbaI and XhoI restriction sites [18]. The construction was sequenced on an ABI 3730 Genetic Analyzer (Applied Biosystems) using the sequencing platform installed at the Oswaldo Cruz Foundation (Fiocruz).

*T. cruzi* epimastigote forms of Dm28c clone (Dm28c-WT) were suspended at 1 × 10^8^ cells/mL in electroporation buffer (EPB) containing 137 mM NaCl; 5 mM KCl; 0.7 mM Na_2_HPO_4_; 6 mM glucose; 21 mM HEPES, pH 7.3. The cellular suspension (400 μl) was mixed with 50 μg of plasmid, placed in a 0.2 cm cuvette and subjected to a pulse of 0.45 kV, 500 μF at room temperature in a Gene Pulser apparatus (BioRad Laboratories) [19]. The parasites were resuspended in LIT medium and stable transformants were selected with 200 to 500 μg/mL of G418. Thereafter, positive epimastigotes were selected by serial dilution in a 96 plate, and selected by bioluminescence emission with SteadyGlo reagent (Promega) in a SpectraMax2 microplate reader.

### *In vitro* bioluminescent imaging

To perform a bioluminescent curve of intracellular Dm28c-luc amastigotes, LLC-MK2 cells (10^3^) were plated in a 96 well microplate and maintained for 18 hours in RPMI 1640 medium with 5% FBS at 37°C in a 5% CO_2_ atmosphere. Afterwards, the cells were washed and allowed to interact with 10^2^ to 10^4^ trypomastigotes of Dm28c-luc. Infected cells were cultivated for 24 to 48 hours in RPMI 1640 medium with 5% FBS at 37°C in a 5% CO_2_ atmosphere. Bioluminescent images were obtained from trypomastigotes, and from intracellular parasites, after 24 and 48 hours of cultivation. Images were acquired after 5 minutes of incubation of trypomastigotes with 150 μg/ml of D-luciferin in 200 μl of RPMI medium in a black 96 well microplate. Intracellular amastigotes were removed from the plate with 50 μl of SteadyGlo reagent (Promega), transferred to the 96 well black microplate and the volume adjusted to 200 μl of RPMI with 150 μg/ml of D-luciferin. Bioluminescence was quantified with automatic measurement ROI.

### *In vivo* bioluminescent imaging

The infection with transgenic parasites was also monitored over time by detecting the bioluminescence in the whole animal using the IVIS Lumina image system (Xenogen). The equipment consists of a cooled charge-coupled camera mounted on a light-tight chamber with a nose cone delivery device to keep the mice anesthetized during image acquisition (IVIS 100; Xenogen). D-luciferin potassium salt (Xenogen) stock solution was prepared in phosphate-buffered saline (PBS) at 15 mg/ml, filter-sterilized, and stored in a-80° freezer. To produce bioluminescence, mice infected with Dm28c-luc and infected with Dm28c-WT received an intraperitoneal injection of D-luciferin stock solution (150 mg/kg). All mice were immediately anesthetized in an oxygen-rich induction chamber with 2% isofluorane, and images were captured after 10 minutes incubation to allow substrate distribution. Mice infected with Dm28c-WT were injected with D-luciferin (150 mg/kg) or PBS and submitted to the same procedure in the induction chamber, but images were acquired just after 1 hour and 24 hours post infection (1 dpi).

Bioluminescent images were obtained after 1 hour, at 1, 4 to 28, and 180 dpi (chronic phase). Bioluminescent images were obtained of mice in dorsal, ventral, and lateral position. Anesthesia was maintained during the entire imaging process by using a nose cone isofluorane-oxygen delivery device in the light-tight chamber.

To acquire the bioluminescent images, some parameters were considered based on the level of bioluminescent emission, such as the time of exposure, which ranged from a few seconds to 5 minutes, the number of pixels, and the field of view (FOV) with automatic focus. The Living Image software automatically co-registered the luminescent images, which were taken in darkness and displayed in pseudo-colors that represent the intensity of the signal and the photographic image, generating an *overlay* image.

### *Ex vivo* bioluminescent imaging

Mice infected with 1 × 10^5^ Dm28c-luc trypomastigote forms were euthanized at 14, 21, 28, and 180 dpi (6 months), and selected organs were excised. The bioluminescence in infected tissues and organs was evaluated in a 24-well plate or in individual plates in 150 μg/ml D-luciferin diluted in enough volume of PBS, to cover the tissues, 0.5 ml for 24 well plate and 1 to 2 ml in individual plates. Images were captured using the IVIS Lumina image system (Xenogen), after 5 minutes incubation with substrate.

### *In vivo* and *ex vivo* bioluminescent image quantification

To measure photon radiance, regions of interest (ROI) were selected on the surface of the mice or the 24-well plate using the automatic ROI. Bioluminescence was measured quantitatively by the Living Image® software, which gave the total flux of photons or radiance (photons/second from the surface) in each pixel, summed or integrated over the ROI area, in a square centimeter (cm^2^) of the tissue. These data were multiplied by one steradian (sr). The photon radiance is displayed as the average radiance, which is the sum of the radiance from each pixel inside the ROI/number of pixels or superpixels (photons/sec/cm^2^/sr) and the standard deviation of the pixel radiance inside the ROI. Bioluminescence, acquired by the CCD camera, was quantified by the Living Image® software using three types of ROI: (1) the automatic ROI measurement tool, which identifies bioluminescent emission automatically, considering a threshold of 20 to 28%; (2) the average background ROI, which measures the background signal in the area specified by the user and corrects the bioluminescent emission by subtraction; and (3) the subject ROI, which identifies each animal in a image. Whenever necessary, individual bioluminescent spots (foci) were summed to get the photon radiance measurements (photons/sec/cm^2^/sr) of each region of the animal: abdomen, thorax, paws, snout, ears, and urogenital region.

In the plate, bioluminescence acquired by the CCD camera was obtained with two types of ROI, the automatic measurement ROI, using a threshold of 25%, and the average background ROI. After subtraction of background signal, bioluminescent radiance measurements (photons/sec/cm^2^/sr) of spots distributed in the organ were summed to get the total radiance measurement of each organ: heart, rectum, gonads, fat tissue, intestines, tail, ears, snout, hind limbs, and forelimbs. Thereafter, the sum of bioluminescent radiance found in the whole 24-well plate, considered 100%, was used to estimate the percentage of bioluminescence in each organ.

### Histological analysis

Mice infected with Dm28c-luc, with Dm28c-WT and uninfected control mice, were euthanized at the time points indicated in the figure legends to collect heart, rectum, gonads, fat tissue from gonadal depot, intestines, tail, ears, snout, hind limbs, and forelimbs to be processed as described elsewhere [20]. Briefly, fragments were fixed using Millonig-Rosman solution [21], and 5 μm-thick slices of paraffin-embedded samples were further processed and stained using hematoxylin-eosin (HE). To obtain different and representative regions of tissues, two 5 μm-thick slices in an interval of 80 μm were collected per slide. Qualitative analysis of tissues was based on cellular inflammatory infiltration and parasite nests.

### Cytokine assays

Blood from mice infected with Dm28c-luc, with Dm28c-WT and uninfected control mice, was obtained by cardiac puncture using 3.8% sodium citrate and plasma was separated after centrifugation, 1000 g/10 minutes at 4°C, for cytokine analysis by flow cytometry (BD FACSCalibur™ flow cytometer) using the Cytometric Array kit (FlowCytomix Mouse Th1/Th2 10plex) (eBiosciences, San Diego, CA). This is a bead-based detection system for quantitative detection of GM-CSF, IFN-γ, IL-1α, IL-2, IL-4, IL-5, IL-6, IL-10, IL-17, and TNF-α. The evaluation was carried out according to manufacturer instructions. Statistical analyses were performed using the Excel software. Data from cytokine analyses of Dm28c-WT and Dm28c-luc were compared using a two-tailed unpaired *t-*test. Differences were considered significant if *p* values were < 0.05.

## Results and discussion

### Longitudinal evaluation of *T. cruzi* infection in mice

We showed in a previous publication that the genetically modified bioluminescent epimastigote forms of Dm28c-luc were stable after two years of cultivation in medium and were able to differentiate into metacyclic trypomastigote forms that infected LLC-MK2 cells *in vitro*. Transgenic trypomastigote forms of Dm28c-luc, derived from cell culture, showed stable expression for six months (the period tested) [12], allowing longitudinal evaluation of the acute and chronic phases of infected mice.

**Figure 1:**
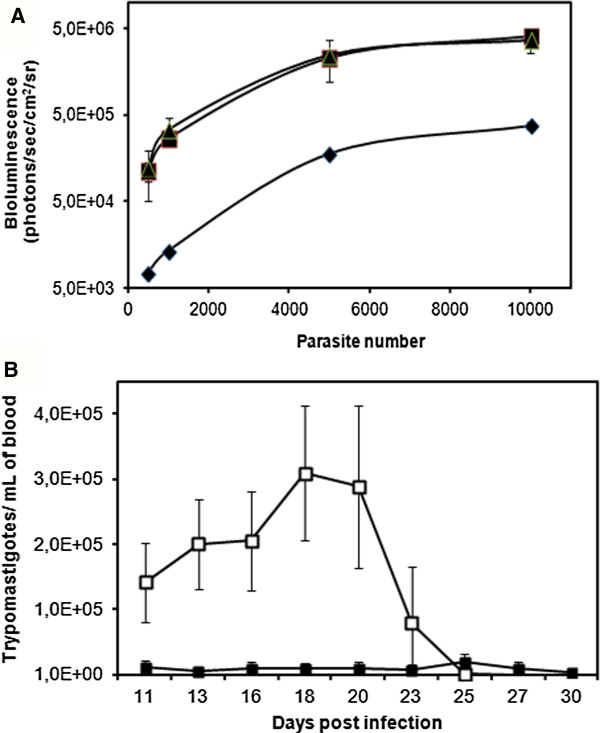
***In vitro *and *in vivo *parasite density curve. (A)***In vitro* bioluminescent measurement in the IVIS lumina system. Dm28c-luc trypomastigotes (diamond); and intracellular parasite, after 24 hours (square) and 48 hours (triangle) post infection. Three independent experiments were performed. **(B)** Parasitemia in mice infected with different *T. cruzi* populations. Animals were infected with 10^5^ trypomastigotes of Dm28c-WT (open squares, n = 10), or Dm28c-luc (solid squares, n = 15). Two independent experiments were performed.

To demonstrate that luciferase expression is stable in all biological forms of transgenic parasites, Dm28c-luc trypomastigotes were allowed to differentiate back to epimastigotes. Bioluminescent emission was in the range of epimastigotes maintained in LIT medium [12], displaying 20643 ± 1495 relative luminescence unit (RLU), as evaluated in the microplate reader, Spectra Max2. For intracellular amastigote evaluation, we used LLC-MK2cells previously infected with 10^2^ to 10^4^ trypomastigotes. Thus, Dm28c-luc trypomastigotes and LLC-MK2 cells infected for 24 and 48 hours were evaluated for bioluminescent emission using the IVIS Lumina system (Xenogen). Bioluminescence could be significantly detected in at least 1 × 10^3^ trypomastigotes and intracellular parasites could be detected in LLC-MK_2_ cells infected with as few as 5 × 10^2^ trypomastigotes (Figure 1A), non infected LLC-MK_2_ was used as negative control. Indeed, bioluminescence in infected LLC-MK_2_ was 10 fold higher than bioluminescence in trypomastigotes used to infect LLC-MK_2_ (Figure 1A). Moreover, evaluation of *in vitro* infection of LLC-MK_2_ cells by optical microscopy of stained cells, suggests that Dm28c-luc parasites have lower infection rates: (1) after 24 hours, 20 to 10% for Dm28c-luc and 16 to 27% for Dm28c-WT; (2) after 48 hours, 9 and 9.4% for Dm28c-luc and 10 to 18% for Dm28c-WT. But once differentiated into amastigotes, these cells retain normal proliferation, comparable to wild type parasites, which ranged from 1.1 to 1.3 amastigotes per infected cell, for Dm28c-WT and Dm28c-luc (n = 2).

As a first step to characterize infection in mice using transgenic Dm28c-luc parasites, we compared blood parasitemia with that of mice infected with the Dm 28c-WT. As shown in Figure 1B, mice infected with the wild type strain showed a typical parasitemia curve for Dm28c, which increased up to approximately 20 dpi (3.0 ± 1.0 × 10^5^ trypomastigotes/mL) and then gradually decreased. Parasitemia peak and magnitude is in accordance with previous publications [22,23]. However, in mice infected with Dm28c-luc trypomastigote forms we found lower levels of parasitemia (Figure 1), but those mice showed active infection with patent levels of circulating parasites at all time points. This observation indicates that the course of infection may vary between Dm28c-WT and Dm28c-luc parasites and indicates that caution may be taken in the interpretation of results obtained with transgenic parasites.

**Figure 2:**
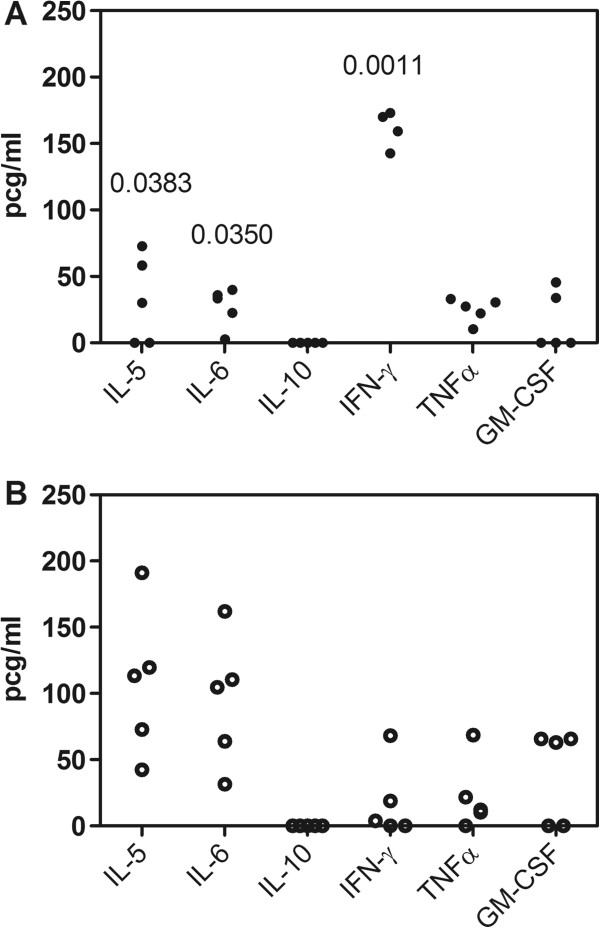
**Analyses of cytokines in the acute phase of infection.** Comparison of cytokine expression in the blood of mice infected with Dm28c-WT **(A)** and Dm28c-luc **(B)** 20 dpi. Statistically significant differences were observed for IL-5, IL-6, and IFN-γ when the *p* value was <0.05 (*t-*test).

Blood cytokines evaluated on 20 dpi, showed no detectable levels of IL-1α, IL-2, IL-4, IL-17 (data not shown), or IL-10 (Figure 2) in mice infected with Dm28c-WT and Dm28c-luc strains of *T. cruzi*. Dm28c-WT strain induced low levels of IL-5, IL-6, TNF-α, and GM-CSF, but high levels of IFN-γ (Figure 2), when compared to uninfected control mice (data not shown). High levels of IFN-γ have been described in the acute phase of the infection, mainly produced by NK and CD4^+^ T lymphocytes [24]. Interestingly, the infection with Dm28c-luc induced a different profile of cytokines, with lower levels of IFN-γ and increased levels of IL-5 and IL-6 (Figure 2). IL-6 is an important inflammatory cytokine, while IL-5, on the other hand, is a T cell-derived cytokine that mainly promotes proliferation, activation, and differentiation of eosinophils. Indeed, the inflammatory process and hindpaw edema caused by the Dm28c-luc transgenic parasite does not reproduce the natural infection (personal communication, Julio Scharfstein). It is possible that genetic manipulation for transgenesis also affected the expression and/or immunogenicity of antigens of the parasite, which led to an altered immunological response. It would be interesting to make a cytokine kinetic analysis of the blood to evaluate the profile of infection with Dm28c-luc throughout the acute and chronic infection, but this is not the scope of the present study.

**Figure 3:**
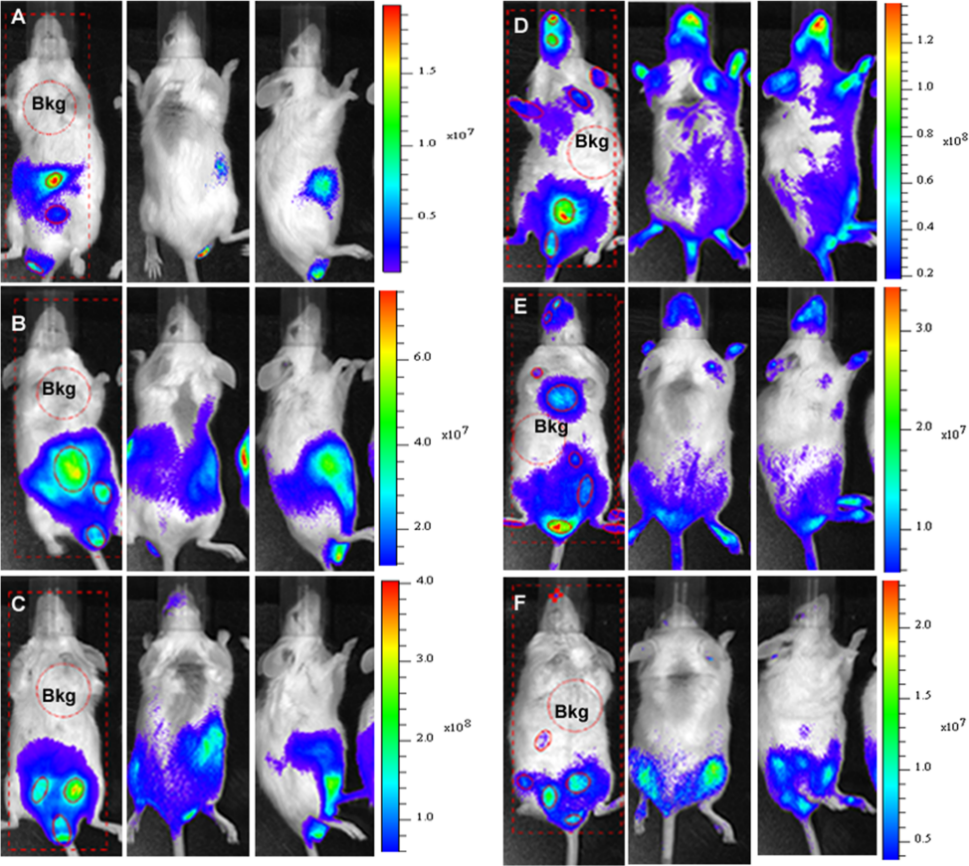
**Follow-up of BALB/c mice infected with 10**^**6 **^**trypomastigotes of transgenic Dm28c-luc strain.** Bioluminescent images were acquired for the same group of mice and one mouse is shown in the ventral (left panel), dorsal (central panel), and lateral (left panel) position after 10 minutes of D-luciferin injection. The same mouse was evaluated after 1 dpi **(A)**; 4 dpi **(B)**, 7 dpi **(C)**, 14 dpi **(D)**, 22 dpi **(E)**, 29 dpi **(F)**. The settings were binning small (small pixels), 10 seconds exposure. For bioluminescent signal quantification, images of mice in the ventral position were evaluated. Subject ROI (square) was used to identify each individual in the image, average background ROI (circle, red dots) measured the background bioluminescent signal which was used to correct the signal in the automatic measurement ROI (red line, circles). Scale bar of bioluminescent image radiance (photons/sec/cm^2^/sr) of mouse in ventral position.

**Figure 4:**
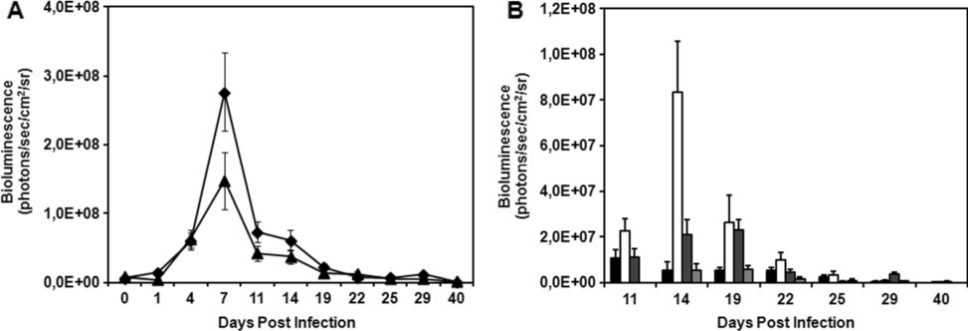
**Follow up of BALB/c mice infected with 10**^**6 **^**trypomastigotes of transgenic Dm28c-luc strain.***In vivo* quantification of bioluminescent foci were performed in images of mice in the ventral position after subtracting the background signal from measurement ROI, identified in the images by the automatic ROI tool (n = 5). Average and standard deviation of radiance, in the abdomen (diamond) and urogenital region (triangle) **(A)**, thorax (black square), snout (white square), paws (dark gray square), and ears (gray square) **(B)**.

To initiate longitudinal follow-up, BALB/c mice were intraperitoneally infected with 1x10^6^ trypomastigote forms of Dm28c-luc. The same group of animals was evaluated for a month, until the beginning of the chronic phase of disease. Before each data acquisition, groups of three animals were placed in the same position inside the IVIS lumina chamber and were imaged in ventral (Figure 3–left panels), dorsal (Figure 3–central panels), and lateral (Figure 3–right panels) position. One hour after infection, bioluminescence was observed in the peritoneal cavity, near the site of inoculation, and in the urogenital region (not shown). Twenty-four hours post inoculation (1 dpi), the abdomen displayed an increase in the bioluminescent signal as a whole (Figure 3A–left and right panels). The urogenital region showed prominent bioluminescence that was more visible in the lateral position (Figure 3A–right panel). This pattern was observed until 7 dpi (Figures 3B,C–all panels, and Figure 4A), when bioluminescence reached a peak in the urogenital and abdominal regions (Figure 3C–all panels, and Figure 4A). On 7 dpi, bioluminescence was also observed in the dorsal region and in the snout of the mice (Figure 3C–central panel). On 11 dpi, the infection had disseminated to other regions of the body, as seen via bioluminescent imaging (not shown) and its quantification (Figures 4A and B). On 14 dpi, foci of bioluminescence were found distributed in different regions of the head, including the nose and ears, and in the tail and paws (Figure 3D–all panels, Figures 4A and B). Bioluminescent foci were also detected in the thorax (cardiac region) (Figures 3D and 4B). However, the abdomen displayed a decreased bioluminescent signal, which was even lower on 22 and 29 dpi (Figures 3E and F, Figure 4A). At 22 dpi, a peak of bioluminescence in the thorax (heart) of some mice was noticed (Figure 3 E–left panel). Around 29 dpi, bioluminescence began to decline in regions of the body and was restricted to certain foci (Figure 3F–all panels, Figure 4B) such as the urogenital region (Figure 3 F–all panels, Figure 4A).

### *In vivo* and *ex vivo* evaluation of acute and chronic phases of *T. cruzi* infection

**Figure 5:**
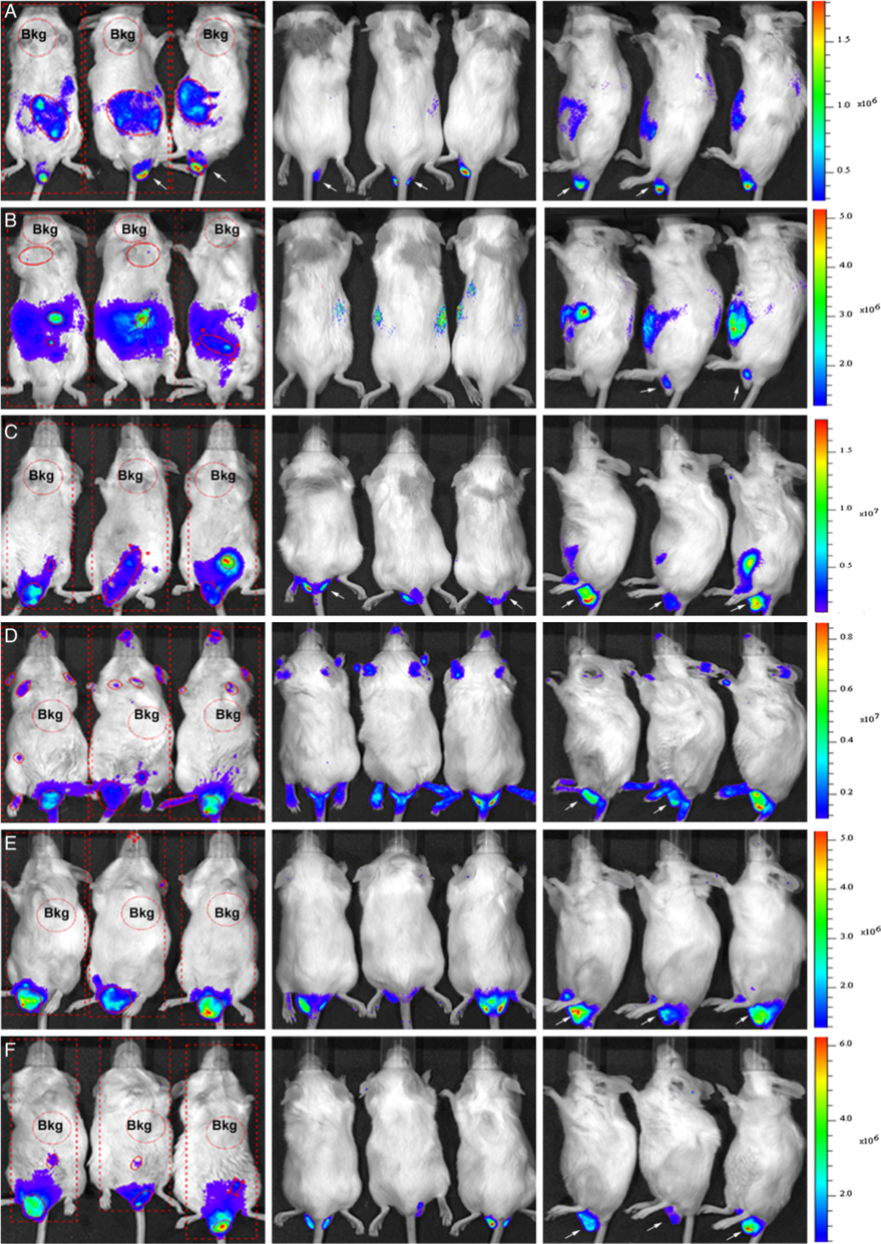
**Follow-up of BALB/c mice infected with 10**^**5 **^**trypomastigotes of transgenic Dm28c-luc strain.** Bioluminescent images were acquired for the same group of mice, three mice are shown in the ventral (left panel), dorsal (central panel), and lateral (left panel) position after 10 minutes of D-luciferin inoculation. The same three mice were evaluated one hour post infection 0 dpi **(A)**; 1 dpi **(B)**, 7 dpi **(C)**, 14 dpi **(D)**, 21 dpi **(E)**, 28 dpi **(F)**. The settings were binning medium (medium size pixels), 1 minute exposure. For bioluminescent quantification images of mice in the ventral position were evaluated. Subject ROI (square) was used to identify each individual in the image, average background ROI (circle, red dots) measured the background bioluminescent signal which was used to correct the signal in the automatic measurement ROI (red line, circles). Arrows indicate urogenital region. Scale bar of bioluminescent image radiance (photons/ sec/ cm^2^/sr) of mice in ventral position.

**Figure 6:**
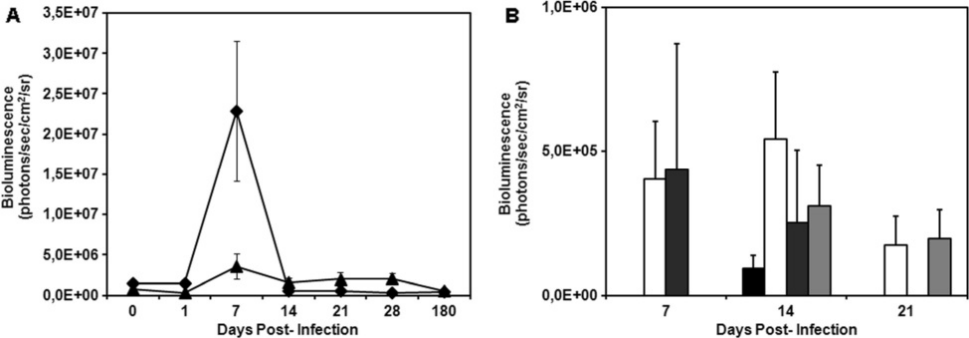
**Follow up of BALB/c mice infected with 10**^**5 **^**trypomastigotes of transgenic Dm28c-luc strain.***In vivo* quantification of bioluminescent foci were performed in images of mice in the ventral position after subtracting the background signal from measurement ROI, identified in the images by the automatic ROI tool (n = 12). Average and standard deviation of radiance of abdomen (diamond) and urogenital region (triangle) **(A)**, thorax (black square), snout (white square), paws (dark gray square) and ears (gray square) **(B)**. Dpi 0 corresponds to measurements made 1 hour after inoculation.

**Figure 7:**
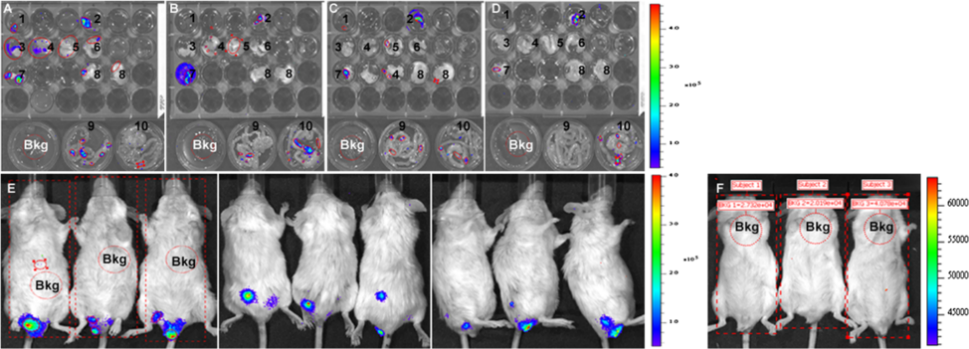
**Evaluation of *T. cruzi *infection by *ex vivo *and *in vivo *bioluminescent imaging of chronic infection.** For *ex vivo* evaluation of organs, images were acquired after incubation with D-luciferin in PBS in a plate, the settings were 5 minutes exposure and binning medium (pixels size). For *in vivo* evaluation, bioluminescent images were acquired in ventral (left panel), dorsal (central panel), and lateral (left panel) position after 10 minutes of intra-peritoneal inoculation of D-luciferin. The settings were binning small (small pixels), 1 minute exposure. 14 dpi **(A)**, 21 dpi **(B)**, 28 dpi **(C)**, 180 dpi *ex vivo***(D)**, 180 dpi *in vivo***(E)** and negative control **(F)**. 1–heart, 2–rectum, 3–snout, 4–testicle and adipocyte, 5–forepaw, 6–hindpaw, 7–tail, 8–ear, 9–small intestine, 10–large intestine. For bioluminescent signal quantification, subject ROI (square) identified the animal in the image of ventral position, average background ROI was placed in the animal or in the plate, on the liver, (Bkg, circle in red dots) to measure the background bioluminescent signal which was used to correct the signal in an automatic measurement ROI (red line, circle), to produce a background-corrected bioluminescent signal. Scale bar of bioluminescent image radiance (photons/ sec/ cm^2^/sr).

**Figure 8:**
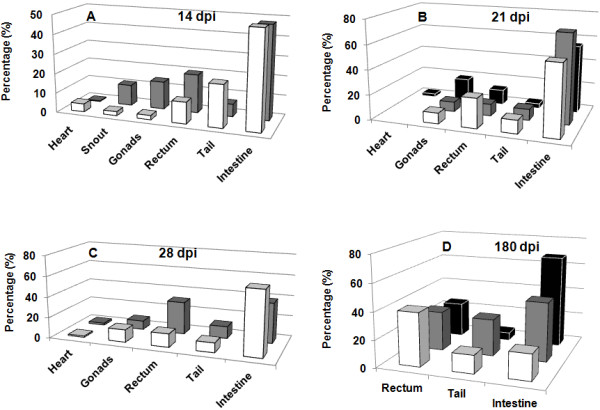
**Follow-up of BALB/c mice infected with 10**^**5 **^**trypomastigotes of transgenic Dm28c-luc strain.***Ex vivo* quantification of bioluminescent foci, identified in dissected organs in the plate, after subtracting the background signal (liver) from measurement ROI, identified in the images by the automatic ROI tool. Heart, snout, gonads,rectum, tail, and intestine. 14 dpi **(A)**; 21 dpi **(B)**, 28 dpi **(C)**, and 180dpi **(D)**. Mice 1 (white square), mice 2 (gray square), mice 3 (black square). Percentage of bioluminescent radiance in each organ is related to total bioluminescent radiance found in the whole 24-well plate.

To evaluate the development of the pathology with this new methodology, mice were also infected with a lower parasite load of 1 × 10^5^ trypomastigotes of Dm28c-luc (n = 20) or Dm28c-WT (n = 20) strain. Each group of mice infected with Dm28c-luc was evaluated by bioluminescent imaging on 0, 1, 7, 14, 21, 28 and 180 dpi, until euthanized for e*x vivo* evaluation of selected organs. The same pattern of infection was observed via bioluminescence in the abdomen, with subsequent migration of trypomastigotes to the urogenital region 1 hour (0 dpi) after inoculation (Figure 5A–all panels; Figure 6A). Twenty-four hours after infection (1 dpi), bioluminescence was disseminated into the peritoneal cavity (Figure 5B), and the urogenital region displayed a lower bioluminescent signal (Figure 6A). In some mice a punctuate site of infection, below the gonads, was observed, which in some cases corresponded to the terminal region of the rectum and portions of the large intestine (Figure 5B–left panel). One week after infection (7 dpi), two bioluminescent peaks were observed: (1) at the abdominal region; and (2) at the urogenital region (Figure 5C, left and right panels; Figure 6A). Some dispersed foci of infection, however, were observed in other regions of the body (Figure 6B). In the second week (14 dpi), infection was distributed to regions of the body, as evaluated by migration of bioluminescent parasites to the head, ears, paws, and tail (Figure 5D–all panels) and measurements of acquired bioluminescent signal (Figure 6B). However, the intensity of bioluminescence in the urogenital and abdominal region was reduced at 14 dpi (Figure 6A). Considering that the diffusion of light through the tissues provides a blurred image, which prevents the precise identification of organs that are infected, we dissected groups of mice to confirm the precise location of infection. On 14 dpi three mice were euthanized, the organs were collected and incubated with substrate D-luciferin for *ex vivo* evaluation in the IVIS® Imaging System. Organs of each mouse were collected and grouped in one plate, bioluminescent radiance was found in the whole 24-well plate, considered as 100%, this was used to estimate the percentage of bioluminescence in each organ. About 50% of bioluminescence was concentrated in the intestine, large and small intestines, and 30% was distributed through the rectum, in the base of the tail, gonads, and fat tissue (Figure 7A; Figure 8A). We also observed 4% to 10% of bioluminescence in the snout of dissected mice (Figure 8A). In one animal we observed approximately 4% bioluminescence in the heart (Figure 8A).

*In vivo* evaluation on the third week (21 dpi) demonstrated that bioluminescence was reduced in the thorax, snout, ears, paws, and abdomen (Figure 5E–all panels; Figures 6A and B), but the urogenital region displayed stable bioluminescence (Figure 6A). *Ex vivo* evaluation of infected organs by bioluminescent imaging demonstrated that the colon, cecum, and rectum displayed patches of bioluminescence that corresponded to 60% of bioluminescence found in the organs investigated in the plate (Figure 7B; Figure 8B), followed by the testicles and tail, which displayed 20% of bioluminescent emission; the heart showed 3% (Figure 8B). On the fourth week (28 dpi) post inoculation, bioluminescence declined overall in the body (Figure 5F–all panels, Figure 6A). *Ex vivo* evaluation revealed that infection was mainly distributed along the intestines and rectum, followed by the gonads and tail (Figure 7C, Figure 8C). Chronically infected mice, 180 dpi, displayed some foci of infection in the urogenital region, which could be quantified (Figure 6A and Figure 7E–all panels). To show that bioluminescent emission is directly related to mice infected with Dm28c-luc, images of mice infected with Dm28c-WT were included as negative control (Figure 7F). *Ex vivo* evaluation of dissected mice corroborated that bioluminescence was predominant in the intestine, followed by the rectum and tail (Figure 7D, Figure 8D). In other organs, such as the heart, bioluminescence was undetected. Therefore, the *ex vivo* evaluation supports the analysis made *in vivo* and shows the precise sites of infection in the urogenital region. The same profile of infection, with distribution of bioluminescent parasites for some regions of the body and its containment to the urogenital region, was observed in BALB/c and Swiss mice (data not shown).

### Genetically modified Dm28c-luc versus Dm28c-WT strain

**Figure 9:**
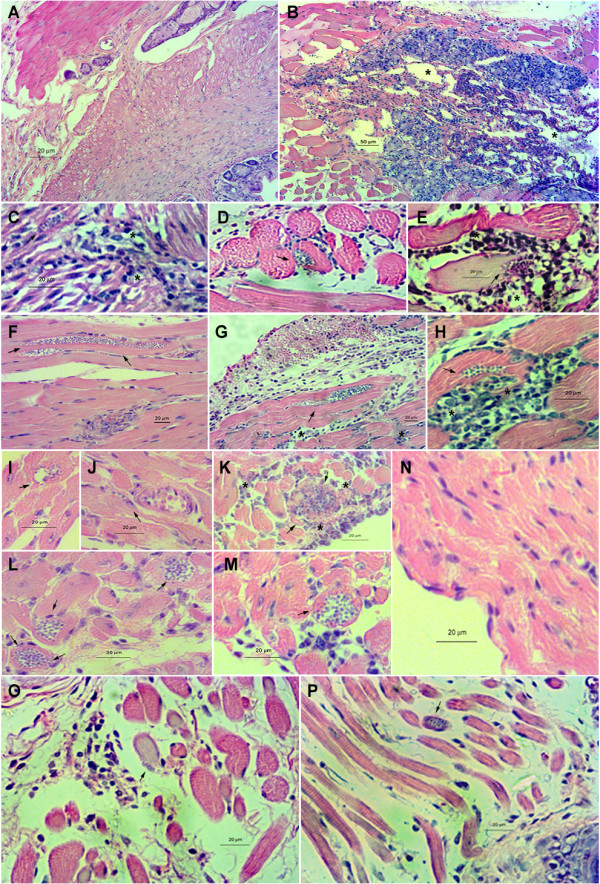
**Parasite localization in tissues of infected animals.***Ex vivo* evaluation of amastigote nests in the rectum and anal region **(A-H)**, heart **(I-N)** and in the snout **(O-P)** in BALB/c mice infected with 10^5^ trypomastigotes of Dm28c-luc or Dm28c-WT. Rectum of uninfected mice, control **(A)**. Rectum of mice infected with transgenic Dm28c-luc stain **(B-E)**: 14 dpi **(B-C)**, 21 dpi **(D)**, 28 dpi **(E)**. Rectum of mice infected with Dm28c-WT strain **(F-H)**: 14 dpi **(F)**, 21 dpi **(G)**, 28 dpi **(H)**. Heart of mice infected with transgenic Dm28c-luc stain **(I-K and N)**: 14 dpi **(I-J)**, 21 dpi **(K)** 28 dpi **(N)**. Heart of mice infected with Dm28c-WT **(L-M)**: 14 dpi **(L)** and 21 dpi **(M)**. Snout of mice infected with transgenic Dm28c-luc **(O)** and Dm28c-WT strain **(P)**. Arrows indicate amastigote nests and asterisks inflammatory infiltrate.

After *ex vivo* evaluation of dissected organs by bioluminescent imaging, the tissues were embedded in paraffin, stained with hematoxylin and eosin, and observed under light microscopy (Figure 9). It was possible to observe the pattern of Dm28c-luc and Dm28c-WT migration, supporting the bioluminescent imaging and demonstrating the presence of amastigote nests in the terminal region of the intestine (Figure 9B-H). Amastigote nests were observed mainly in the anal region of mice infected with both strains, Dm28c-WT or Dm28c-luc (Figure 9B-H). However, oversized amastigote nests were observed on 14 dpi in the rectum and anal region of mice infected with Dm28c-WT (Figure 9F) as compared to Dm28c-luc (Figure 9B-C). From 21 to 28 dpi, the nests observed in the terminal region of the intestine of mice infected with Dm28c-WT decreased in size and in number of parasites (Figure 9G-H). However, with Dm28c-luc parasites, amastigote nests were more evident on the 28 dpi, which suggest a delay in the development of infection in the rectum and anal region of mice infected with Dm28c-luc (Figure 9D-E). We also observed inflammatory foci in close proximity to parasite nests in the tissues infected with both strains, Dm28c-luc (Figure 9B) or Dm28c-WT (Figure 9F). However, regions of intense lymphocyte infiltrate were observed mostly in mice infected with Dm28c-WT (Figure 9F-H). As described previously, myenteric denervation has been observed in the acute phase of *T. cruzi* infection. It seems that the mechanism involves IFNγ, iNOS activation in the inflammatory foci in the intestine, and NO production [25]. Therefore, in the infection with transgenic Dm28c-luc, the reduced levels of IFNγ and lymphocyte infiltrate could reflect a less deleterious effect in the colon and rectum.

Independent of strain tropism, previous publications demonstrated a decline in intestinal mobility, evaluated by charcoal elimination, in mice infected with different *T. cruzi* strains, such as Dm28c, CL-Brener, Brazil, and TcY. In the same work, it was demonstrated that Dm28c interferes with intestinal mobility earlier than do other strains, in the first week of infection [22].

In our study, the alterations observed in tissues of rectum and anal sphincter are compatible with an inflammatory process that could lead to disruption of the nervous system; however, other tissues, glands, and regions of the intestine (not investigated) could also be sites of infection, as observed with naturally infected *Opossum*[26]. Thus, the novel foci of infection in the acute and chronic phases, such as (a) the rectum, (b) the anal sphincter, (c) the testicle and its associated adipocyte tissues, and (d) the base of the tail, could also have an impact in the maintenance of infection in sylvatic, peri-domestic, and domestic cycle and in the transmission of the disease by routes not explored previously. In the case of *Opossum*, contamination of environment and food with parasites from the anal gland region has been shown [26]. Adipose tissue was described as playing a role as a reservoir for recrudescence of infection [5].

Considering that the heart is one of the most studied and affected organ by *T.cruzi* infection, we performed also an *ex vivo* evaluation of the heart by bioluminescent imaging coupled to histological analysis, and compared the myocardium infection caused by Dm28c-WT and Dm28c-luc. In mice infected with Dm28-luc, bioluminescent emission could be detected until 28 dpi with a peak at 14 dpi, and by histological analysis a few amastigote nests and scattered inflammatory cell infiltrates could be observed at 14 dpi (Figure 9I and J). However, in mice infected with Dm28c-WT, heart tissues showed more amastigote nests and a few inflammatory infiltrates (Figure 9L). In the third week after infection (21 dpi), a few and small inflammatory infiltrates dispersed in the cardiac fiber of mice infected with Dm28c-luc were observed, some of them with amastigotes (Figure 9K). However, in mice infected with Dm28c-WT a few but clearly defined amastigote nests and inflammatory infiltrate were observed (Figure 9M). At 28 dpi no amastigote nest and inflammatory infiltrate was found, only discrete and scattered inflammatory cells were observed in mice infected with Dm28c-WT or Dm28c-luc (Figure 9N).

In the murine model, previous studies have demonstrated the evolution of experimental *T. cruzi* infection in heart tissues, with the presence of extracellular matrix components and inflammatory infiltrate in the myocardium, caused by Y strain in the acute phase of the disease. In opposition, infection caused by Dm28c strain failed to display inflammatory infiltrate and showed rare amastigote nests [23], which suggested that different strains could display different heart tissue responses [27,28].

In order to find other sites of parasite reservoirs and infection, we evaluated tissues from the snout region such as the cheeks and nose of infected mice by histopathology analysis. In accordance with bioluminescent imaging, on 14 dpi, parasite nests were also observed in the snout, in muscle fibers of mice infected with each strain, Dm28c-WT (Figure 9O) or with Dm28c-luc (Figure 9P). Considering the organization of the tissue, which was composed of collagen and connective tissues, it was more difficult to evaluate the size of nests and to find parasite nests on 21 and 28 dpi. It is important to point out that in spite of the small size of amastigote nests in the muscle fibers of the snout, bioluminescent emission was above the threshold and displayed enough photons to be captured and quantified, which confirms that firefly luciferase expression in Dm28c-luc is stable and suitable for bioluminescent image studies.

Although amastigote nests were considerably more frequent in animals infected with Dm28c-WT, the Dm28c-luc strain was able to infect mice and displayed the same pattern of infection. *In vivo* and *ex vivo* bioluminescent imaging associated with histological analyses of tissue sections corroborated that amastigote nests were larger and more frequent in the rectum and anal region, if compared to the nests found in the heart and other tissues, and these sites of infection remained during the chronic phase.

Another aspect that should be considered about bioluminescent imaging is that the methodology is able to identify even small foci of infection in the organ, but depending on the level of luciferase expression and the tissues infected, the bioluminescent signal can be under the detection limit. Due to the limitations of histological techniques to evaluate residual and small foci of infections in tissues and organs, additional techniques such as PCR and qPCR should be considered to evaluate the presence of parasites in tissues and correlate bioluminescent image with parasite burden.

Previous work has shown the use of bioluminescent imaging *in vivo* to follow the parasite expressing firefly luciferase within their mammalian host [29], and developed a high-throughput bioluminescent imaging assay for drug trials in mice inoculated with *T. cruzi* in the hind footpads [30]. It was observed that the progression of infection in mice infected with CL strain by intra peritoneal inoculation was similar to our study, displaying a peak at 10 to 14 dpi, but *ex vivo* bioluminescent imaging was restricted to a few organs [29]. Thus, we performed longitudinal evaluation with quantification of bioluminescent foci *in vivo* coupled to a detailed *ex vivo* analyses of several regions and organs until the chronic stage of infection, 180 dpi. Our study gives new information and insights about the progression and distribution of infection throughout tissues and foci that are not usually explored but are important sites of infection, and should be considered in studies with murine models. If this pattern of infection is also related to the strain Dm28c or to the site of inoculation, it is the scope of our next work.

The biological and genetic variability of *T. cruzi* has led to its classification into six groups or discrete typing units (DTU) [31,32]. In this context, Dm28c is classified as TcI; this strain was isolated from *Didelphis marsupialis* in Venezuela, and the TcI genotype is widespread in Colombia, Venezuela, and Central America, displaying emerging importance [33,34]. Genotype analysis of isolates from chagasic patients, domestic and wild mammals, and triatomine bugs in Venezuela demonstrated that this group of *T. cruzi* accounted for most of the isolates [35]. However, the distribution of infection throughout the new foci of infection such as, (a) the rectum, (b) the anal sphincter, (c) testicles and (d) its fat tissues, is usually not evaluated in the population affected by Chagas disease and during treatment, but can have a role in disease prognosis.

## Conclusion

The present work demonstrated that bioluminescent imaging associated with microscopic examination of tissue sections is a powerful technology that can be used for evaluation of infection in the murine model.

The genetically modified strain is stable, and suitable for follow-up studies *in vivo* in the murine model, whose intensity of bioluminescent emission increases in accordance with the inoculum used to infect—10^6^ (Figures 3A and B) or 10^5^ (Figures 5A and B). The fact that the transgenic strain could be less lethal, but able to evolve into the chronic phase for 6 months to one year (not shown), can be an advantage for drug trials *in vivo*. This methodology brought, for the first time, new information about the progression and dynamics of infection, detecting new sites of infection.

## Competing interests

The authors declare that they do not have any competing interests.

## Authors’ contributions

The author WS idealized the project, contributed to the experimental design and manuscript preparation. CH produced the strain, executed bioluminescent imaging, data analysis and manuscript elaboration. AHP performed cytokine analysis and the result interpretation, contributed with histopathology analysis. MMB and AS performed histological techniques and analysis and contributed with the manuscript. All authors read and approved the final version of the manuscript.
